# Progress in Neurology

**Published:** 1903-11-14

**Authors:** 


### Progress in Neurology.

**Surgical Treatment of Brain Tumours.**[1]—Starr has collected from medical literature the statistics of the results of operation in cases of tumour of the brain. The figures are very interesting. They are as follows:—

| | Cerebral. | Cerebellar. |
|---|---|---|
| Total number of cases operated on | 315 | 50 |
| Cases in which the tumour was not found ... ... ... ... | 91 | 20 |
| Cases in which the tumour was found but not removed ... ... | 21 | 6 |
| Cases in which the tumour was removed and the patient died ... | 51 | 8 |
| Cases in which the tumour was removed and the patient recovered | 152 | 16 |

The percentage of recoveries in cerebral cases was thus 48 per cent., in cerebellar cases 32 per cent. It is, however, practically certain that not all the unsuccessful cases are reported. The accuracy of diagnosis is to-day more easily possible than it was. Given a history of a slowly progressive disease with symptoms of headache, vomiting, vertigo, sensations of cerebral discomfort, progressive emaciation and feebleness, and a gradual loss of sight due to a choked disk, the only probable hypothesis is the existence of a brain tumour. Diagnosis of its position is wholly by direct observation of local symptoms such as (*a*) mental symptoms; (*b*) motor or sensory aphasia; (*c*) local spasm and monoplegia; (*d*) hemianopsia; (*e*) cerebellar staggering. The absence of any one of these localising symptoms makes it evident that the tumour is inaccessible. The chance of success has been materially increased by the newer surgical methods, *e.g.* the electrical saws. The causes of failure are: mistaken diagnosis of the location of the tumour, which are sometimes inevitable, for local symptoms are not infallible in their indication; secondly, some tumours even when accessible cannot be removed, *e.g.* infiltrating gliomata; thirdly, the dangers of hæmorrhage and the dangers of meningitis.

**Opium Habit.**—In a paper on the opium habit, Jelliffe [2] gives details of the bromide method of cure, which is one of the very best, though like all others it is only possible when the patient is confined to rooms and under surveillance; 120 grains of sodium bromide are given in half a tumbler of water every two hours during the daytime until one ounce has been administered in the same day. This may be sufficient to produce the "bromide sleep," or the drug may have to be continued on the third day. It is a safe rule after 24 hours if the drowsiness is so profound that the patient cannot be roused from sleep to take further doses, or when aroused is incoherent. There is usually some difficulty in feeding the patient, swallowing being sometimes impossible, so that rectal alimentation is necessary. Any septic condition of the pharynx or naso-pharynx or mouth should contraindicate the method, and a weak heart or impaired pulmonary conditions are absolute contra-indications. Jelliffe says that this method is of marked value in well selected cases. His experience of the use of hyoscine is contradictory, for this, though of good service in stages of marked excitement is apt to produce dangerous shock. Halleck [3] has had good results in the treatment of the morphia habit by cutting off the drug absolutely and at once and giving a combination of strychnine $\frac{1}{60}$ gr., hyoscine $\frac{1}{200}$ gr., and codeia $\frac{1}{4}$ gr. It is found that the strychnine stimulates all the functions and increases the appetite, whilst the nausea, restlessness, insomnia and pain are cared for by the hyoscine and codeia. The celerity of cure, with no suffering to speak of, and the very small amount of medicine used, is certainly remarkable. The cases may relapse, but they are cured at any rate for the time.

1 Jour. of Nerv. and Mental Dis., July, 1903. 2 Amer. Jour. Med. Sci., May, 1903. 3 Med. Rec., April 11, 1903.

(*To be continued.*)

